# Hybrid deep feature and machine learning framework for classification of thyroid nodules in ultrasound images

**DOI:** 10.3389/fonc.2026.1784411

**Published:** 2026-05-25

**Authors:** Dingnan Zhang, Bo Li, Hao Ju, Tingxue Li, Yanzhu Zhang

**Affiliations:** 1 School of Automation and Electrical Engineering, Shenyang Ligong University, Shenyang, China; 2 Department of Ultrasound, Shengjing Hospital of China Medical University, Shenyang, China

**Keywords:** computer-aided diagnosis, deep transfer learning, machine learning, medical image classification, thyroid nodules, ultrasound imaging

## Abstract

**Introduction:**

Accurate differentiation between benign and malignant thyroid nodules is essential for reducing unnecessary biopsies and improving early clinical decision-making. This study aims to enhance the reliability of ultrasound-based thyroid nodule assessment by proposing an optimized computer-aided diagnosis (CAD) framework.

**Methods:**

A hybrid CAD framework combining deep transfer learning with gradient-boosted decision tree classification was developed. High-level semantic features were extracted from a pretrained ResNet50 model and encoded as fixed-length representations. These features were then fed into the CatBoost algorithm for downstream classification, enabling the framework to leverage both the representational richness of deep neural networks and the decision efficiency of boosted tree ensembles, especially under the condition of limited annotated medical data. A comprehensive set of complementary evaluation measures was used to assess the diagnostic performance, which reflects overall accuracy, error distribution, and the model’s ability to distinguish clinically meaningful malignant cases.

**Results:**

Experimental results demonstrate that the proposed framework, which couples transfer-learned deep features with CatBoost, achieves superior discrimination between benign and malignant thyroid nodules compared with conventional approaches. It exhibits improved robustness across variations in ultrasound appearance and maintains stable performance without the need for extensive parameter tuning.

**Discussion:**

These findings highlight the potential of the proposed method as an efficient and reliable tool for computer-aided thyroid nodule diagnosis. They also underscore the framework’s suitability for integration into real-world clinical workflows, which could further optimize clinical decision-making and reduce unnecessary medical interventions.

## 1 Introduction

Thyroid cancer is the most common malignant tumor of the endocrine system, accounting for approximately 90% of all endocrine neoplasms (1). In recent years, its global incidence has been on a continuous upward trend, a phenomenon evident across various regions worldwide (2). In terms of disease characteristics, although most thyroid cancers exhibit relatively indolent biological behavior with slow progression and favorable patient prognosis, the incidence and mortality rates of some advanced lesions and highly aggressive pathological subtypes are still rising, posing a severe threat to patients’ life and health (1). These epidemiological and disease outcome changes highlight the importance of accurately identifying malignant thyroid nodules at the early stage of the disease and intervening in diagnosis and treatment in a timely manner, which is also a key link to improve the overall prognosis of patients.

Ultrasonography has become the preferred imaging modality for clinical screening of thyroid nodules due to its high popularity, low examination cost, absence of ionizing radiation, and ability to clearly display various morphological features of thyroid nodules, such as border morphology, internal echo, and calcification status (3). To improve the diagnostic consistency among medical institutions and physicians with different levels of experience, the American College of Radiology developed the ACR TI-RADS system. This system integrates several core ultrasound indicators, including nodule border clarity, echo intensity characteristics, aspect ratio, and calcification patterns, establishing a standardized risk stratification framework for thyroid nodules and providing a unified reference standard for the preliminary clinical judgment of benign and malignant nodules.

However, the ACR TI-RADS system still has obvious limitations in practical clinical application. On the one hand, the diagnostic efficacy of this system is highly dependent on the personal experience of the operating physician. There are often cognitive differences among different physicians in the interpretation of subtle features in ultrasound images and the consideration of indicator weights, which leads to significant discrepancies in diagnostic results among observers, and the overall diagnostic process is highly subjective (4). On the other hand, in daily clinical work scenarios, ultrasound physicians usually face great time pressure and heavy workload. Long-term continuous operation is prone to cause distraction of physicians’ energy, which will further affect the accuracy of nodule feature interpretation and reduce the consistency of diagnostic results (5).

The limitations of traditional ultrasound diagnostic models have fully highlighted the urgent needs in clinical diagnosis and treatment: there is an imperative to develop auxiliary diagnostic tools with higher objectivity, repeatability, and reliability to compensate for the deficiencies of manual interpretation. In the current field of ultrasound-assisted diagnosis of thyroid nodules, diagnostic schemes that rely solely on a single artificial intelligence (AI) technology have gradually revealed significant shortcomings: while a single deep learning model can efficiently mine deep semantic features of ultrasound images, including morphological information of microcalcification and blurred boundaries, its training is highly dependent on large-scale annotated datasets, and it has insufficient generalization ability for rare nodule subtypes in clinical practice, typical of highly aggressive microcarcinomas (6); the feature extraction capability of a single machine learning model is limited to manually preset ultrasound indicators, such as aspect ratio and echo intensity. It should be noted that such models can neither capture the hidden subtle pathological features in images nor effectively cope with complex noise interference in ultrasound images, which is consistent with the findings that traditional machine learning relies on manual feature engineering and is prone to being affected by image quality factors (7).

To address the above existing problems, this paper proposes an AI model integrating deep learning with traditional machine learning as a more efficient ultrasound-assisted diagnostic tool for thyroid diseases. This model helps clinicians break through the limitations of traditional manual image interpretation and risk stratification, distinguish benign and malignant thyroid nodules more accurately and efficiently, and provide a more solid basis for the formulation of subsequent diagnosis and treatment plans.

Although hybrid combinations of deep feature extraction and machine learning classifiers have been explored in medical image analysis, their application to pathology-linked thyroid ultrasound classification remains insufficiently investigated, particularly in limited-data clinical scenarios. In this study, the methodological contribution does not lie in introducing a completely new backbone architecture, but in constructing and evaluating a practical hybrid framework that couples transfer-learned ResNet50 representations with CatBoost classification for thyroid nodule diagnosis. Compared with end-to-end deep models trained directly on relatively small datasets, this design aims to reduce overfitting while preserving rich semantic image features. Compared with conventional machine learning based on handcrafted descriptors, it avoids reliance on manually engineered ultrasound features and provides a more flexible representation of lesion morphology and texture.

The main contributions of this study are as follows. First, we propose a hybrid ultrasound classification framework that combines transfer-learned deep semantic features with gradient-boosted decision-tree classification. Second, we evaluate whether CatBoost can provide a robust downstream classifier for deep ultrasound features under limited-sample conditions. Third, we compare the proposed framework with several conventional machine learning baselines and discuss its potential role and limitations in computer-aided thyroid nodule assessment.

Given the limited size of the available clinical ultrasound dataset, we adopted a hybrid strategy that combines transfer learning-based deep feature extraction with machine learning classification, with the aim of reducing overfitting and improving robustness under data-constrained conditions.

## 2 Related works

Deep learning algorithms have commonly been used for the differential diagnosis between benign and malignant thyroid nodules (8). Such as the excitation network and squeeze, extreme version of inception and residual neural network (ResNet) (9) (10). They not only overcame the interference caused by the Radiologist, but also exhibit increased resistance to noise and low image qualities (11). These research results show that the accuracies of deep learning models do not significantly statistically differ from senior doctors in the diagnosis of thyroid nodules (9) (10).

For example, Peng et al. developed the ThyNet model and demonstrated the potential of deep learning to assist thyroid nodule diagnosis and management in a multicohort setting. 12) (13) Similarly, Wei et al. reported an ensemble deep learning framework for multicenter thyroid nodule classification, highlighting the feasibility of deep models across heterogeneous ultrasound data. (14) Koh et al. further evaluated a deep convolutional neural network for thyroid nodule diagnosis on ultrasonography and compared its performance with that of expert radiologists. 15).

Traditional ultrasound assessment and TI-RADS-based interpretation remain central to thyroid nodule evaluation, yet they are fundamentally limited by subjectivity and operator dependence. Even though TI-RADS provides a structured scoring system, observers may differ in how they interpret morphological criteria such as margins, echogenicity, and suspicious calcifications (16), and these inconsistencies contribute to diagnostic variability, particularly among less experienced clinicians (5).

To overcome the limitations of morphology-based evaluation, multimodal ultrasound technologies have been introduced. Elastography provides quantitative stiffness information that improves the diagnostic utility of conventional gray-scale ultrasound (17), but its measurement stability is influenced by equipment variations and physiological factors such as carotid pulsation, resulting in limited reproducibility and reduced applicability in routine clinical settings (18). Contrast enhanced ultrasound (CEUS) offers vascular and perfusion insights, yet its diagnostic performance is challenged by the heterogeneity of TI-RADS 4 nodules. These lesions often show overlapping or atypical enhancement patterns, making the interpretation prone to error (19).

Prior thyroid ultrasound studies have already explored hybrid transfer-learning pipelines. For example, Liu et al. combined transfer-learned deep representations with hybrid feature design for thyroid nodule classification in ultrasound images, supporting the feasibility of integrating pretrained deep features with non-end-to-end decision modules in this domain (20). More broadly, hybrid medical-imaging models that combine ResNet-based representation learning with downstream decision modules have shown advantages over standalone ResNet baselines in some settings. For example, Ozturk et al. reported that a DecisionTree-ResNet50 hybrid model outperformed standalone ResNet50 for small intracranial cyst detection, suggesting that hybrid designs may improve robustness in challenging clinical imaging tasks (21).

Although multimodal ultrasound improves diagnostic information to some extent, the dependence on operator technique, equipment variation, and subjective interpretation remains difficult to eliminate. These constraints have motivated the development of computational diagnostic tools that leverage medical imaging data to provide objective, consistent, and reproducible assessments—forming the rationale for the automated classification framework proposed in this study.

## 3 Data collection and dataset description

We collected ultrasound images from patients who visited a Grade A tertiary hospital between January 2022 and July 2025. All of them had a definite pathological diagnosis of the nodules. All the nodules were examined by the same radiologist. who has over 10 years of experience in imaging and over 5 years of experience in needle biopsy.

A total of 390 thyroid ultrasound images were included in this study, comprising 130 benign images and 260 malignant images. The benign-to-malignant ratio was therefore 1:2, indicating a moderate class imbalance in the dataset. All nodules had definitive reference diagnoses established by fine-needle aspiration cytology and/or postoperative histopathology. Nodules classified as Bethesda II were considered benign, whereas nodules classified as Bethesda V or VI were considered malignant. Bethesda III–IV nodules were excluded unless postoperative pathology was available to provide a definitive diagnosis.

The study inclusion criteria were as follows: firstly, pathological findings obtained with surgical resection or fine-needle aspiration biopsy due to thyroid nodules, secondly, ultrasound examination was performed within a month before surgery or FNA, and thirdly, the ultrasound images are clear and the clinical data are complete. The exclusion criteria were as follows: patients who received radiofrequency or microwave ablation, chemotherapy or radiotherapy before ultrasound examination, and the puncture pathology failed to provide a clear diagnosis and no surgical treatment was received. Due to the retrospective nature of the study the requirements for informed consent were waived.

**Figure 1:**
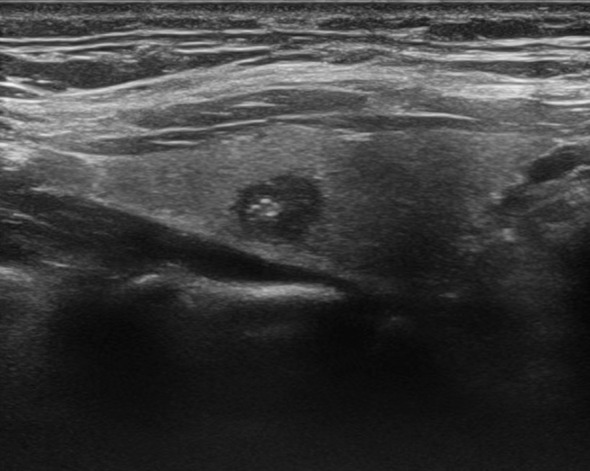
Representative examples of raw ultrasound images.

Thyroid nodule examinations were performed using multiple clinical ultrasound systems, including Canon Aplio 500, and Mindray Resona R9G, Philips IU22, SuperSonic Aixplorer V, each equipped with high-frequency linear probes operating between 7–15 MHz. The use of diverse ultrasound machines ensured heterogeneity in image appearance, which contributes positively to the generalizability of the proposed learning framework. Representative examples of the raw ultrasound images collected are shown in **Figure 1**.

All images were initially stored in JPG format, from which both the pixel matrix and metadata were extracted. To ensure standardized input across all imaging platforms, the JPG images were converted to PNG format while preserving the original spatial resolution. Each thyroid nodule was linked to a definitive clinical label based on cytological examination (Bethesda classification) or postoperative histopathology, which served as the ground truth. Nodules classified as Bethesda II were considered benign, while nodules classified as Bethesda V or VI were labeled malignant. Bethesda III–IV nodules were excluded from the dataset unless there is a post-operative pathology report for these nodule to avoid ambiguity in diagnostic certainty.

All the nodules were examined by the same radiologist. who has over 10 years of experience in imaging and over 5 years of experience in needle biopsy. For each image, the radiologist provided a bounding box surrounding the nodule and assigned the corresponding benign/malignant label. This multi-stage annotation procedure ensured the reliability and consistency of the labels used in model training.

## 4 Materials and methods

**Figure 2:**
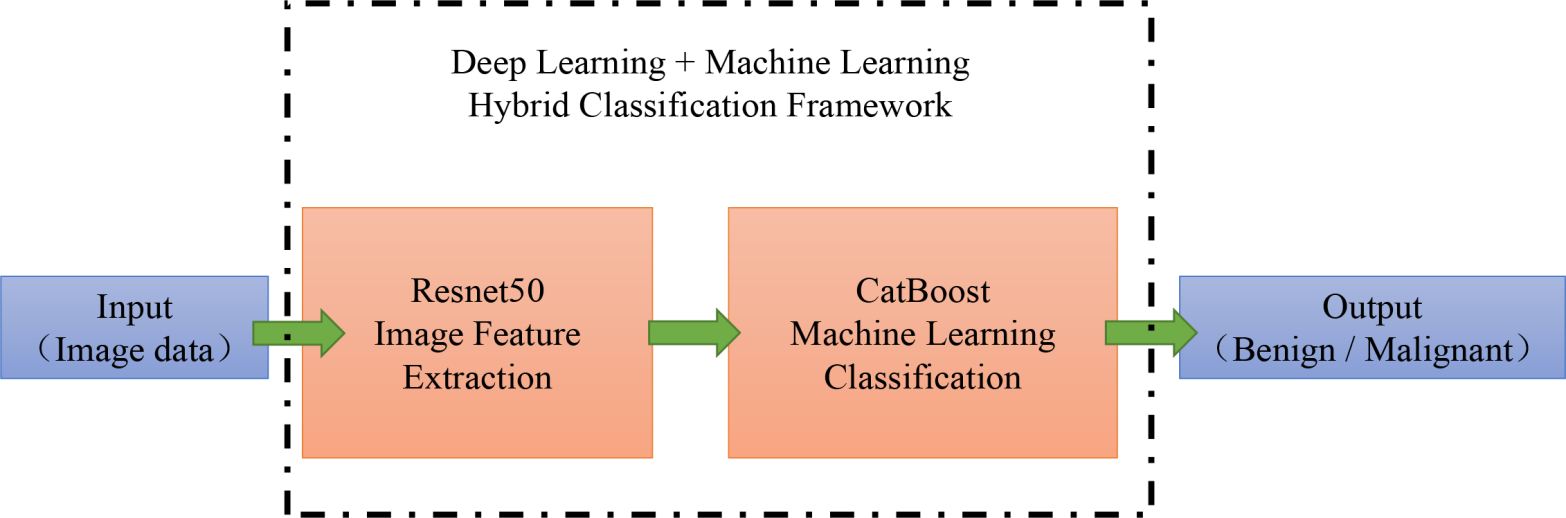
The overall workflow of the proposed method.

The purpose of this study is to develop an accurate and reliable computer-aided diagnosis (CAD) system capable of distinguishing benign and malignant thyroid nodules from ultrasound images. Based on the limited number of clinical ultrasound images available in our dataset, we adopt a hybrid classification architecture that strategically integrates transfer learning-based deep feature extraction and ensemble machine learning classifiers. The rationale for this architecture lies in leveraging CNNs’ powerful image representation ability while avoiding overfitting through limited parameter training, thus enabling the model to generalize under small-sample medical scenarios. The overall workflow of the proposed method is illustrated in **Figure 2**, consisting of three sequential modules: image preprocessing, deep feature extraction via a pretrained ResNet-50 network, and benign/malignant classification using tree-based machine learning algorithms.

### 4.1 Image preprocessing and augmentation

Following annotation, all ultrasound images underwent preprocessing to normalize the data distribution and reduce irrelevant variations. Each cropped nodule image was resized to 224 × 224 pixels to maintain compatibility with the ImageNet-pretrained ResNet50 backbone.

**Figure 3:**
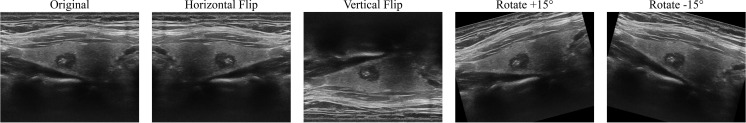
Examples of augmentation images.

To improve model robustness and reduce overfitting, several data augmentation strategies were applied during training. These augmentations simulated common variations encountered in clinical ultrasound imaging. The operations included random horizontal flipping, small-angle rotations within ±10°, adaptive gamma correction to capture intensity variability, and the addition of Gaussian noise to mimic speckle patterns. Examples of augmentation images are shown in **Figure 3**, which summarizes the transformation steps applied to each training sample.

The use of augmentation increased the diversity of the dataset, enriched intra-class variability, and allowed the learning algorithms to better capture the intrinsic characteristics of thyroid nodules independent of imaging artifacts.

### 4.2 Overview of the hybrid deep learning–machine learning pipeline

**Figure 4:**
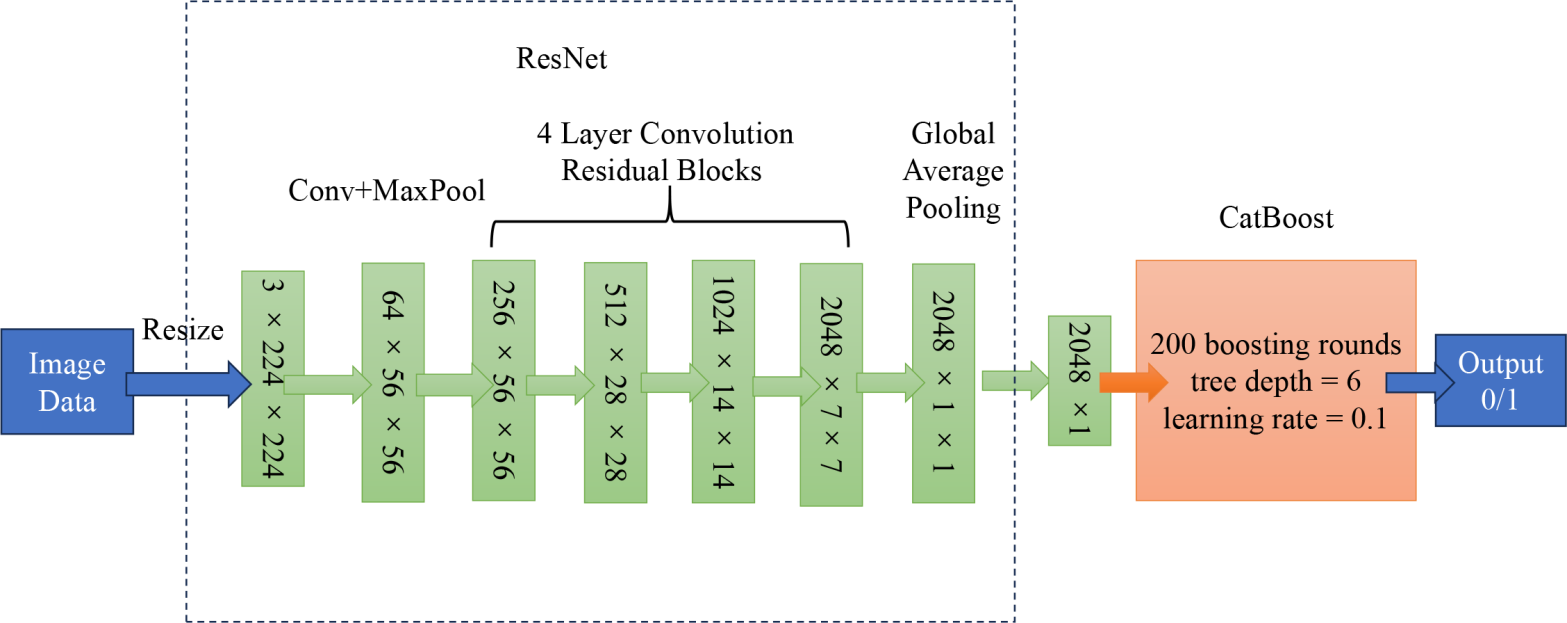
The structure of the hybrid AI-based model.

The overall framework of this study integrates a deep learning–based feature extractor with three machine learning classifiers, forming a hybrid architecture optimized for thyroid nodule classification. A conceptual overview of the pipeline is shown in **Figure 4**.

The pipeline begins with preprocessing, after which all images are forwarded into a ResNet50 network pretrained on ImageNet-1K. The deep network transforms each ultrasound image into a high-dimensional semantic feature vector that captures texture, edge structure, and morphological patterns indicative of nodule malignancy. The extracted feature vectors are then used as input to classifiers based on CatBoost, which outputs a predicted label. These predictions are further aggregated during the evaluation stage to assess model performance.

This hybrid design combines the strong representation learning ability of deep neural networks with the interpretability and robustness of tree-based classifiers. The modular nature of the system also allows for easy replacement or refinement of individual components without modifying the overall architecture.

### 4.3 Deep feature extraction using ResNet50 transfer learning

**Figure 5:**
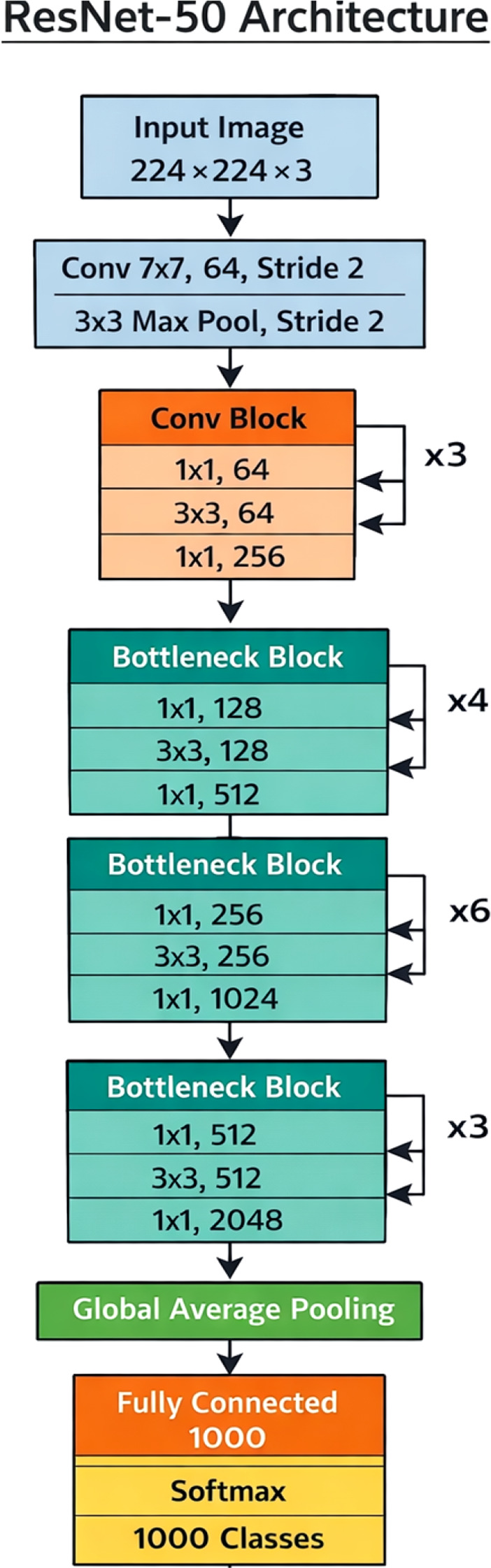
The network structure of ResNet50.

As shown in **Figure 5**, the network architecture of ResNet50 consists of 50 weighted convolutional layers (Conv) and fully connected layers (FC). Different from the Basic Block residual units adopted in shallow ResNets (e.g., ResNet18), this model takes Bottleneck residual blocks as the core building units, and its overall structure can be divided into 6 highly reproducible stages. The specific structure and algorithm design are as follows: the input layer accepts 3-channel input images (with a default size of 224×224×3), which are then processed by an initial convolutional layer (7×7 Conv, 64 convolution kernels, stride=2, padding=3), a Batch Normalization (BN) layer, a ReLU activation function, and a 3×3 max pooling layer (stride=2, padding=1). Subsequently, the processed features pass through Stages 1–4, which are stacked with 3, 4, 6, and 3 Bottleneck residual blocks respectively. For each stage, the shortcut connection of the first Bottleneck uses a 1×1 Conv (stride=2) for dimension adjustment, while the rest adopt identity mapping shortcuts. Finally, the output is generated through global average pooling (GAP) as shown in Equation 1, a 1×1000 fully connected layer (adapted to the classification task on the ImageNet dataset), and a Softmax activation function.

A ResNet50 convolutional neural network pretrained on the ImageNet-1K dataset was employed to extract deep visual features from the ultrasound images. The final fully connected classification layer of the original model was removed, and only the convolutional layers and global average pooling (GAP) layer were retained. This enabled the model to serve purely as a feature extractor rather than an end-to-end classifier.

The input image, with dimensions 3×224×224, passes first through a 7×7 convolutional layer with stride 2, producing a 64×112×112 feature map. This is followed by max pooling, reducing the spatial size to 56×56. Four sequential residual stages—conv2x, conv3x, conv4x, and conv5x—progressively increase the number of channels while reducing the spatial dimensions. The detailed transformation of feature maps across stages is summarized in Table.

The final output of the conv5x stage is a 2048×7×7 tensor. The global average pooling layer compresses this representation into a 2048-dimensional feature vector, denoted as:

(1) $$\text{f}=\text{GAP}\left(\text{ResNet}50_{\text{conv}}\left(\text{I}\right)\right)$$

This vector captures high-level semantic features of the nodule, including heterogeneity, echogenicity patterns, margin characteristics, and microcalcifications. Such features are clinically relevant indicators of malignancy and are effectively encoded through the hierarchical structure of the ResNet50 backbone.

### 4.4 Machine learning classification models

CatBoost, an optimized variant of Gradient Boosting Decision Trees (GBDT), is structurally characterized by symmetric binary decision trees as base learners and constructs a strong learner via an iterative boosting framework that sequentially fits the gradient of the loss function; its core algorithmic innovation lies in the Ordered Boosting mechanism, which mitigates the prediction shift issue induced by sample reordering during traditional GBDT training, while it natively supports end-to-end processing of categorical features without relying on complex preprocessing steps—specifically, it employs Target Statistics (TS) encoding to transform categorical features into numerical statistics, thereby retaining the semantic information inherent in categorical variables and circumventing the curse of dimensionality; additionally, the model integrates multiple regularization strategies, including Newton Gradient estimation, tree complexity constraints, sample subsampling, and feature sampling, which not only accelerate the model’s convergence rate but also effectively suppress overfitting, and such structural and algorithmic designs endow CatBoost with the capability to capture high-dimensional and nonlinear feature patterns, as well as the merits of stable training dynamics and robust generalization performance in practical tasks.

After feature extraction, the 2048-dimensional deep feature vectors were used as inputs to CatBoost machine learning classifiers. This model was selected due to its strong performance on tabular data and its ability to model nonlinear interactions among features.

The CatBoost model employed in this study consisted of 200 trees, each with a maximum depth of 6. The use of bootstrap sampling and feature bagging enhances the generalizability of the ensemble by reducing variance and avoiding overfitting. The decision-making process for each split is based on the Gini impurity, as shown in Equation 2:

(2) $$\text{Gini}\left(t\right)=1-\sum_{k=1}^{2}p_{k}^{2}$$

where *p_k_* represents the proportion of samples from class *k* at node *t*.

CatBoost classifiers were trained using gradient-boosted decision trees with 200 boosting iterations, a tree depth of 6, and a learning rate of 0.1. Both models optimized the logistic loss function, as shown in Equation 3:

(3) $$\text{L}=\sum_{i=1}^{n}[y_{i}\log\left(p_{i}\right)+\left(1-y_{i}\right)\log\left(1-p_{i}\right)]$$

where *y_i_* denotes the ground truth label and *p_i_* represents the predicted probability for sample *i*.

CatBoost additionally incorporates ordered boosting and feature permutation strategies, which mitigate prediction shift and reduce overfitting, particularly when dealing with relatively small datasets such as medical imaging cohorts.

### 4.5 Implementation details

To improve reproducibility, all experiments were conducted using a fixed random seed under the same software and hardware environment. The dataset split was performed at the patient level to avoid information leakage between subsets. During feature extraction, the ImageNet-pretrained ResNet50 backbone was used as a fixed feature encoder after removal of the final fully connected classification layer. The resulting 2048-dimensional feature vectors were used as inputs to the downstream classifier.

For CatBoost, the main hyperparameters were set as follows: number of boosting iterations = 200, tree depth=6, and learning rate=0.1. These settings were selected based on preliminary validation performance while keeping the model complexity moderate to reduce overfitting risk on the limited dataset. Model performance was evaluated using accuracy, precision, recall, F1-score, and confusion matrix analysis on the independent test set.

## 5 Result evaluation

This chapter presents a comprehensive evaluation of the proposed method through a series of quantitative and qualitative experiments. The analysis includes a detailed description of the experimental environment, the metrics adopted for performance assessment, comparisons with baseline models, ablation studies, parameter sensitivity investigations, and visualizations of representative results. All experiments were conducted under consistent settings to ensure fair and reproducible comparisons.

### 5.1 Experimental platform

All experiments were conducted on a workstation equipped with an NVIDIA GPU 5090, an Intel Core Ultra 9 285K Processor, and 128 GB of RAM, running Python 3.9 and PyTorch as the deep-learning backend. The CatBoost library was used for classical machine-learning classification, while scikit-learn supported standard data preprocessing and evaluation procedures. The dataset consisted of ultrasound images of thyroid nodules collected under consistent imaging conditions, with each sample manually annotated as benign or malignant by experienced clinicians. All images were standardized to a uniform resolution prior to model input, and the complete dataset was divided into training, validation, and testing subsets following a fixed split to ensure reproducibility. This setup provided a controlled environment for evaluating the discriminative capability and generalizability of the proposed hybrid model.

### 5.2 Evaluation metrics

To comprehensively assess model performance, several quantitative metrics commonly used in medical-image diagnosis were employed, as shown in Equations 4–7. Accuracy measured the overall correctness of predictions, while precision and recall evaluated the model’s ability to correctly identify malignant lesions without generating excessive false alarms. The F1-score provided a balanced indicator combining precision and recall, particularly useful under class imbalance. Additional insight into the model’s decision behavior was obtained from the confusion matrix, which separates true-positive, true-negative, false-positive, and false-negative predictions. Together, these metrics offered a robust and clinically meaningful assessment of classification quality beyond a single accuracy value.

(4) $$\text{Accuracy}=\frac{TP+TN}{TP+TN+FP+FN}$$

(5) $$\text{Precision}=\frac{TP}{TP+FP}$$

(6) $$\text{Recall}=\frac{TP}{TP+FN}$$

(7) $$\text{F}1\text{-score}=\frac{2TP}{2TP+FP+FN}$$

### 5.3 Confusion matrix analysis

**Figure 6:**
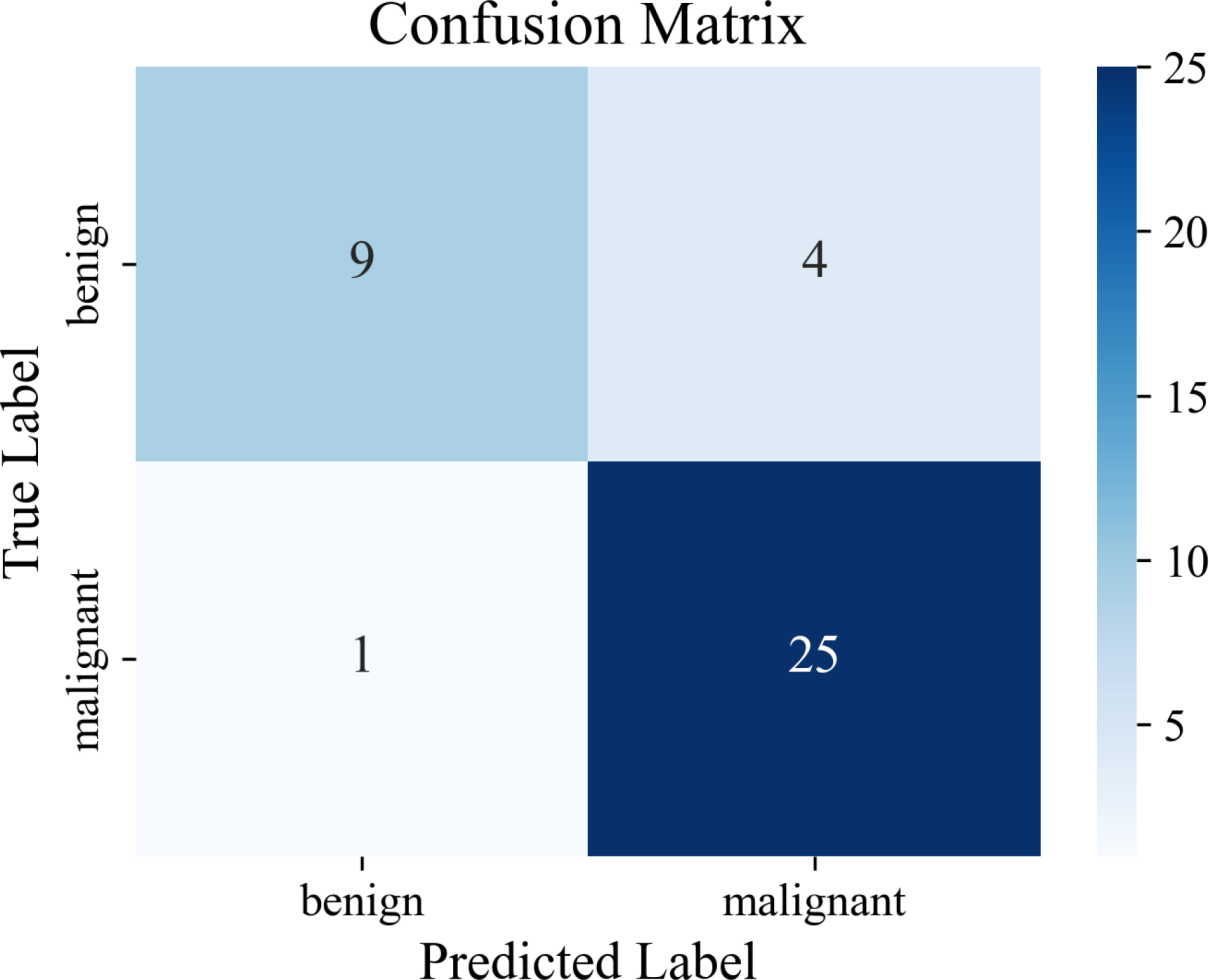
The confusion matrix of ResNet-Catboost model.

The model’s classification behavior on the test set was further evaluated using the confusion matrix shown in **Figure 6**. The results indicate that the ResNet–CatBoost framework achieves a strong true-positive rate for malignant nodules, reducing the likelihood of missed malignancies. Benign cases were also identified with high reliability, although a small number were classified as malignant. This conservative tendency reflects a bias toward minimizing potentially harmful false negatives, a desirable characteristic in clinical decision-support systems for thyroid cancer screening. Overall, the confusion-matrix distribution suggests that the model maintains an effective balance between sensitivity and specificity.

To better reflect the statistical uncertainty associated with the relatively small test set, 95% confidence intervals (CIs) were additionally calculated for the main evaluation metrics. On the independent test set, the proposed model achieved an accuracy of 0.8718 (95% CI: 0.7329–0.9440). When malignant nodules were treated as the positive class, the sensitivity was 0.9615 (95% CI: 0.8111–0.9932), the specificity was 0.6923 (95% CI: 0.4237–0.8732), and the precision was 0.8621 (95% CI: 0.6944–0.9450).

### 5.4 Comparative evaluation across models

**Figure 7:**
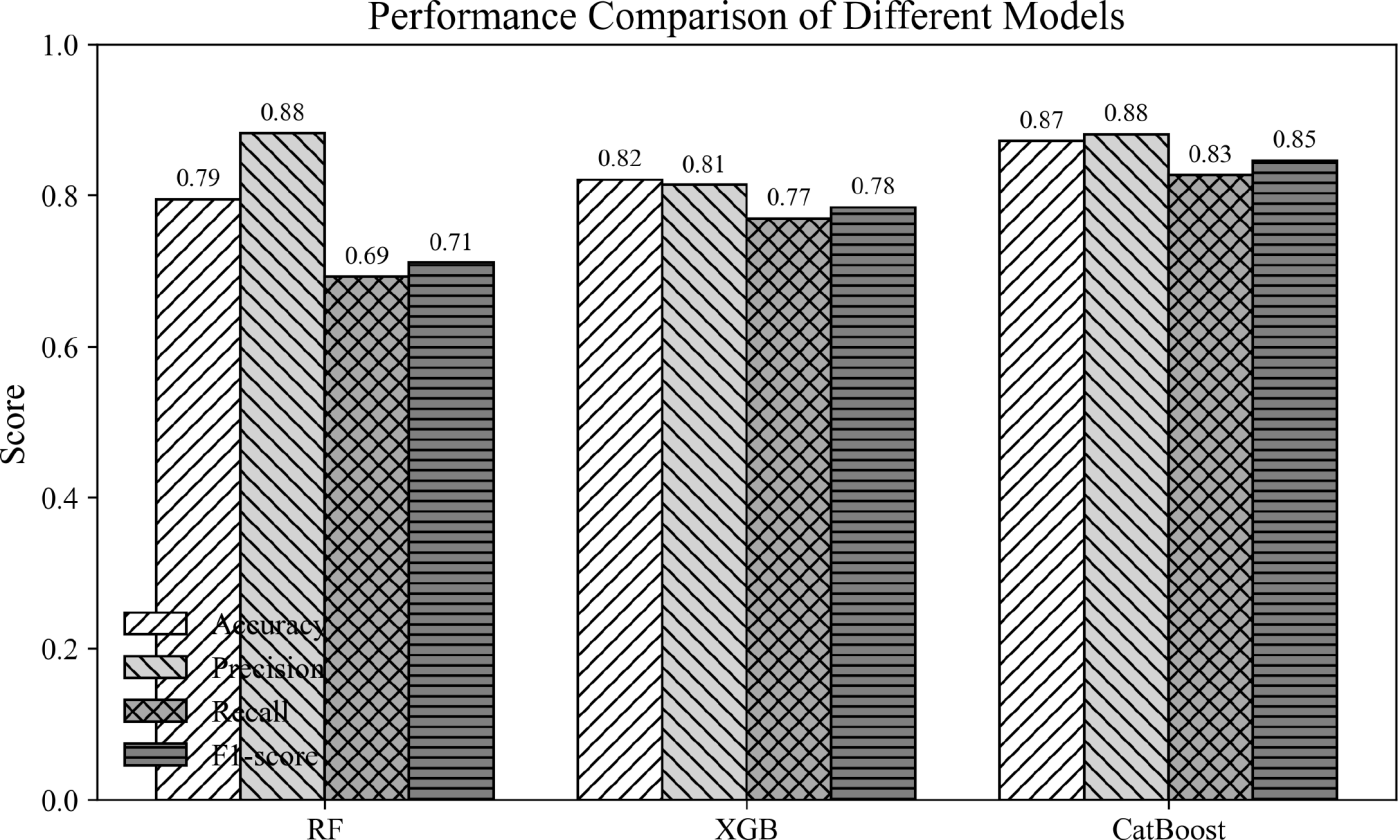
The structure of the hybrid AI-based model.

To validate the advantage of the proposed hybrid model, its performance was compared with alternative classifiers using both deep-learning-based and machine-learning-based approaches. The aggregated results in **Figure 7** show that the ResNet–CatBoost combination consistently achieves superior accuracy, F1-score, and AUC values compared with single-model baselines. The improvement illustrates the complementary strengths of transfer learning and gradient-boosted decision trees: the ResNet backbone provides robust and stable high-level feature representations, while CatBoost effectively models non-linear classification boundaries in limited-data scenarios. This combination results in enhanced generalization and reduced overfitting relative to traditional end-to-end training.

**Figure 8:**
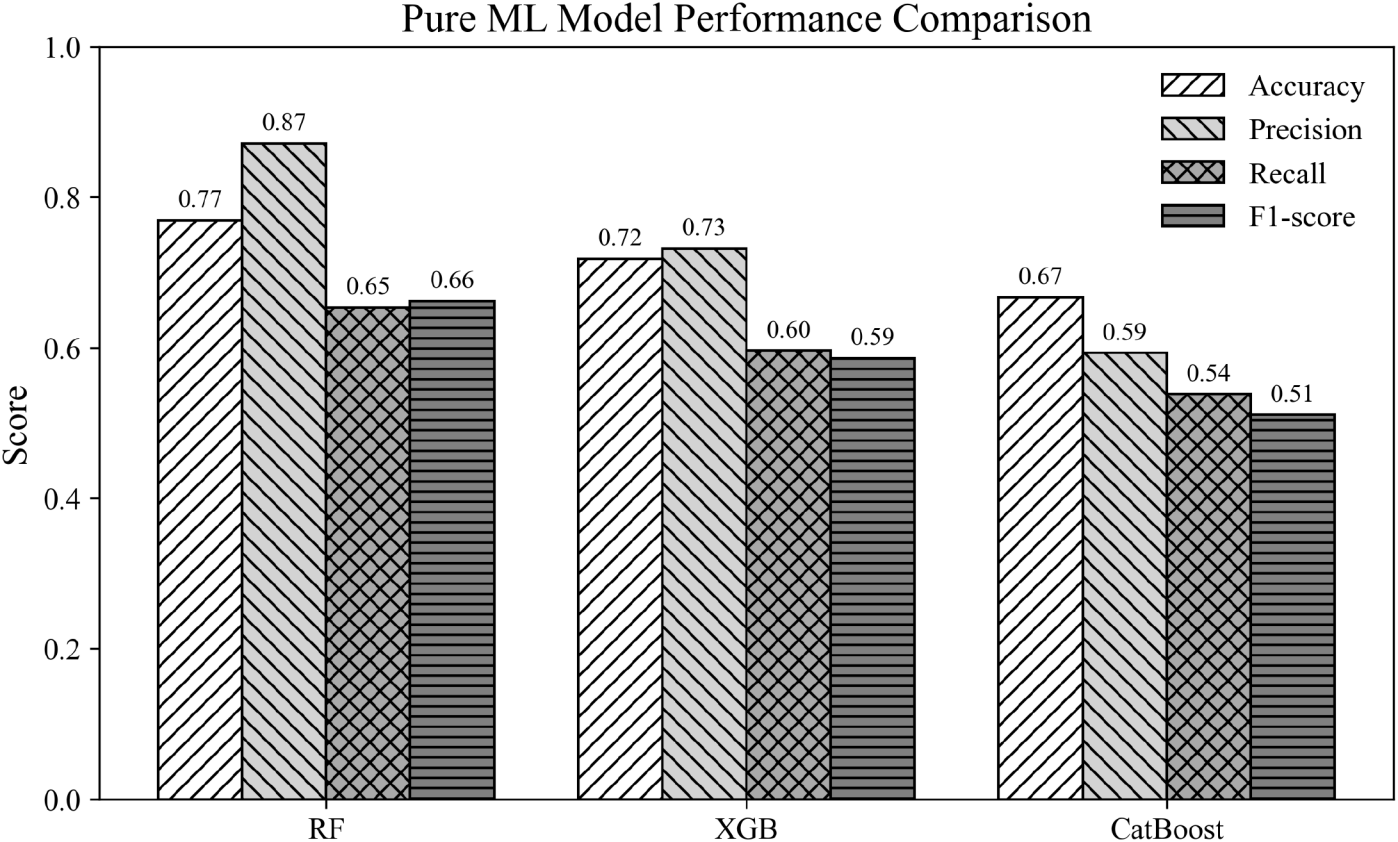
Accuracy comparison of different models.

In addition to comparisons with deep learning models, this study also conducted a series of control experiments based on traditional machine learning methods—using raw image pixels as input features and constructing classifiers with Random Forest, Extreme Gradient Boosting (XGBoost), and Categorical Boosting (CatBoost) respectively—to further verify the impact of the feature extraction quality of the proposed hybrid model on classification performance. The experimental results in **Figure 8** show that, in the thyroid nodule classification task, these pure machine learning methods exhibit significantly inferior performance to the hybrid model, specifically characterized by lower accuracy, poor class separability, and insufficient prediction stability. The abbreviations used in **Table 1** are defined as follows: RF, Random Forest; XGBoost, Extreme Gradient Boosting; CatBoost, Categorical Boosting. **Table 1** presents a comparative analysis of the result data of all models. In contrast, the proposed ResNet-CatBoost hybrid model demonstrates distinct advantages over all pure machine learning methods. This finding fully confirms that the deep features extracted by the hybrid framework play a crucial role in medical image classification tasks.

**Table 1:** Classification performance comparison of different methods on the test set.

Classifier	Accuracy	Precision	Recall	F1-score
RF	0.77	0.87	0.65	0.66
XGBoost	0.72	0.73	0.60	0.59
CatBoost	0.67	0.59	0.54	0.51
ResNet50 + RF	0.79	0.88	0.69	0.71
ResNet50 + XGBoost	0.82	0.81	0.77	0.78
ResNet50 + CatBoost	0.87	0.88	0.83	0.85

## 6 Discussion

The vast majority of thyroid cancers present in the form of nodules. Since early-stage thyroid cancer exhibits almost no symptoms, it is difficult for patients to detect on their own. As a non-invasive examination method, ultrasonography is highly suitable for population-based physical examinations and screening, which also places higher demands on its ability to distinguish between benign and malignant nodules.

Although current mainstream single deep learning-based auxiliary diagnostic algorithms have demonstrated certain application value in medical image analysis, they are limited by the singularity of feature representation, resulting in obvious constraints on diagnostic efficacy and stability in complex clinical scenarios. In view of this, the core innovation of this study lies in breaking through the performance bottleneck of single algorithms by constructing a novel diagnostic model that deeply integrates deep learning and traditional machine learning. Leveraging the powerful deep visual feature extraction capability of deep learning networks and combining it with the superior discriminative ability of machine learning models for high-dimensional features, this model realizes the complementary advantages of the two technical It may help stratify patients at different risk levels and has the potential to assist clinical decision-making; however, its ability to reduce the workload of medical staff or avoid unnecessary fine-needle aspiration biopsies or thyroidectomies still requires further prospective validation. Experimental results suggest that, compared with the evaluated single-model baselines, the hybrid model achieved improved classification performance on the current dataset. However, no direct comparison with sonographers was performed in this study.

A large number of ultrasound images with definite pathological diagnoses were collated and analyzed in this study. All enrolled patients underwent fine-needle aspiration biopsy or surgical operation, among which malignant nodules accounted for 68%. Clinical validation demonstrated that the optimized hybrid model achieves an accuracy rate of 87% in diagnosing malignant thyroid nodules, while there is a certain possibility of misjudgment for benign nodules. This characteristic precisely aligns with the core demand of clinical diagnosis and treatment—prioritizing the guarantee of “zero missed diagnoses”. In other words, it is preferable to classify a small number of benign nodules as suspicious cases rather than miss any potential malignant lesions, thereby minimizing the risk of delayed treatment due to missed diagnosis. Therefore, the hybrid AI-assisted diagnostic tool developed in this study is more suitable for sonographers to diagnose category 4 or above nodules, i.e., it has higher application value for nodules that are difficult for radiologists to distinguish visually.

Theoretically, there is still a possibility of false negatives in category 2 nodules confirmed by fine-needle aspiration biopsy, which may interfere with image processing. To minimize the impact of this issue, experienced radiologists were selected to participate in image annotation in this study, and we plan to further expand the sample size in the future to reduce such interference to a negligible level.

This study also has certain limitations. First, the number of cases included in the study is relatively small. Second, although the physicians involved in puncture and diagnosis have rich clinical experience, it is still impossible to completely avoid the occurrence of supervision bias. In addition, patients independently decide whether to undergo puncture biopsy or surgical operation based on ultrasound results and their own willingness, and this patient selection bias is also a factor that may lead to deviations in the study results.

Another limitation of this study is the class imbalance of the dataset. Specifically, the dataset contained 260 malignant images and 130 benign images, meaning that malignant cases accounted for approximately 66.7% of the total samples. This imbalance may have influenced the model’s classification tendency and the distribution of evaluation metrics. Future work should include a larger and more balanced cohort to further assess the robustness of the proposed framework.

## 7 Conclusion

This study presented a hybrid framework for thyroid nodule classification in ultrasound images by combining transfer-learned ResNet50 features with a CatBoost classifier. The experimental results indicate that the proposed approach can provide promising discrimination between benign and malignant nodules and may outperform several conventional machine learning baselines under limited-data conditions. Nevertheless, the current findings are constrained by the relatively small sample size, retrospective single-center design, lack of external validation, and absence of stronger statistical validation. Future work will focus on expanding the dataset, performing patient-level cross-validation and multicenter testing, incorporating stronger baseline comparisons, and evaluating the model against clinical readers to better assess its practical utility.

## Data Availability

The dataset used in this study consists of ultrasound images of thyroid nodules collected from Shengjing Hospital of China Medical University. Access to the dataset is restricted to research purposes only and may require institutional approval. Requests to access these datasets should be directed to HJ, 18940257518@163.com.
